# Sustained effects on immune cell subsets and autoreactivity in multiple sclerosis patients treated with oral cladribine

**DOI:** 10.3389/fimmu.2024.1327672

**Published:** 2024-02-16

**Authors:** Rikke Holm Hansen, Marina Rode von Essen, Mie Reith Mahler, Stefan Cobanovic, Finn Sellebjerg

**Affiliations:** ^1^ Danish Multiple Sclerosis Center, Department of Neurology, Copenhagen University Hospital - Rigshospitalet, Glostrup, Denmark; ^2^ Department of Clinical Medicine, Faculty of Health and Medical Sciences, University of Copenhagen, Copenhagen, Denmark

**Keywords:** immune reconstitution, multiple sclerosis, cladribine tablets, autoreactive T cell responses, autoreactive B cells

## Abstract

**Introduction:**

Cladribine tablet therapy is an efficacious treatment for multiple sclerosis (MS). Recently, we showed that one year after the initiation of cladribine treatment, T and B cell crosstalk was impaired, reducing potentially pathogenic effector functions along with a specific reduction of autoreactivity to RAS guanyl releasing protein 2 (RASGRP2). In the present study we conducted a longitudinal analysis of the effect of cladribine treatment in patients with RRMS, focusing on the extent to which the effects observed on T and B cell subsets and autoreactivity after one year of treatment are maintained, modulated, or amplified during the second year of treatment.

**Methods:**

In this case-control exploratory study, frequencies and absolute counts of peripheral T and B cell subsets and B cell cytokine production from untreated patients with relapsing-remitting MS (RRMS) and patients treated with cladribine for 52 (W52), 60 (W60), 72 (W72) and 96 (W96) weeks, were measured using flow cytometry. Autoreactivity was assessed using a FluoroSpot assay.

**Results:**

We found a substantial reduction in circulating memory B cells and proinflammatory B cell responses. Furthermore, we observed reduced T cell responses to autoantigens possibly presented by B cells (RASGRP2 and a-B crystallin (CRYAB)) at W52 and W96 and a further reduction in responses to the myelin antigens myelin basic protein (MBP) and myelin oligodendrocyte glycoprotein (MOG) after 96 weeks.

**Conclusion:**

We conclude that the effects of cladribine observed after year one are maintained and, for some effects, even increased two years after the initiation of a full course of treatment with cladribine tablets.

## Introduction

Multiple sclerosis (MS) is an immune-mediated disease characterized by inflammation, demyelination and neuroaxonal damage in the central nervous system (CNS) (1). The etiology of MS remains unknown; however, T (2) and B (3) cells have been implicated in the pathogenesis (4). Specifically, follicular helper T (Tfh) cells, a subset of CD4^+^ T cells expressing CD127, CXCR5, and PD-1, play a critical role in B cell differentiation and antibody production (5–7). In normal immune homeostasis, Tfh cells interact with follicular regulatory T (Tfr) cells, which regulate immune responses induced by Tfh cells (8–10).

Alterations in the balance between Tfh and Tfr cells, favoring Tfh cells (11, 12) and lower Tfr activity (13), have been reported in MS. Additionally, a recent study demonstrated the active recruitment of Tfh cells to the cerebrospinal fluid in MS patients, presumably mediated by the CXC motif chemokine ligand CXCL13, which is associated with intrathecal immunoglobulin synthesis (14).

Tfh cells can be further categorized based on their expression of CXCR3 and CCR6 into Tfh1 (CXCR3^+^CCR6^-^), Tfh17 (CXCR3^-^CCR6^+^), Tfh2 (CXCR3^-^CCR6^-^) (15) and Tfh17.1 (CXCR3^+^CCR6^+^) cells (16), each possessing distinct cytokine production profiles and B cell helper capacities. Furthermore, phenotypically and functionally distinct CD25^-^ and CD25^int^ Tfh cell populations with predominantly Tfh1-like and Tfh17-like phenotypes, respectively, have been identified (14).

In addition to alterations in T cell subsets, abnormalities in circulating B cells have been observed in MS patients. These include increased production of proinflammatory cytokines (17–19) and reduced activity of regulatory B cells (20–22).

The treatment strategy for relapsing-remitting MS (RRMS) includes immune reconstitution therapies, e.g. cladribine tablets (23, 24). Cladribine selectively accumulates in immune cells, where it is phosphorylated to its active form 2-chlorodeoxyadenosine triphosphate (Cd-ATP) by deoxycytidine kinase (DCK), thereby inducing apoptosis (25). The selective effect on lymphocytes is attributed to their high DCK concentrations and low levels of the enzyme 5´-Nucleotidase which inactivates Cd-ATP (25).

Cladribine tablets have demonstrated efficacy as immune reconstitution treatment for RRMS, offering long-term benefits with few treatment cycles (26). Treatment results in reductions of circulating T and B cells, particularly within the memory compartment, without affecting immunoglobulin levels (27–34). Recently, a study from our laboratory showed that one year after the initiation of cladribine treatment, T and B cell crosstalk was impaired in a manner that likely reduced their ability to mediate pathogenic effector functions (35). Additionally, there was a specific reduction of autoreactivity to the autoantigen RASGRP2 which is highly expressed in B cells and brain tissue (35).

Studies of the effect of cladribine on immune cell subsets in year two after treatment initiation are limited. With this study we performed a longitudinal analysis of the effect of cladribine treatment in patients with RRMS, focusing on the extent to which the effects observed on T and B cell subsets and autoreactivity after one year of treatment are maintained, modulated, or amplified during the second year of treatment.

## Methods

### Study participants

In this exploratory case-control study, a total of 30 untreated patients with relapsing-remitting multiple sclerosis (RRMS) and 20 RRMS patients treated with oral cladribine tablets were included. Blood samples were obtained from these patients, which were subsequently used for both *ex vivo* and *in vitro* analysis.

The diagnosis of MS was based on the McDonald 2017 criteria (36). The untreated patients with RRMS were newly diagnosed and had not received any disease-modifying therapy. They were included in the study at least one month after their last steroid treatment.

The administration of a complete cladribine treatment course comprised oral intake of 3.5 mg/kg in two annual courses, each consisting of two treatment weeks, with a four-week interval between. Patients who received cladribine tablets (Mavenclad^®^, Merck KGaA, Darmstadt, Germany) were included in the study at week 52 (W52), i.e., one year after their first annual course. Additionally, they were included at W60, W72, and W96, i.e., 8, 20 and 44 weeks after the beginning of second annual course.

### Study protocols approval, registrations, and patient consent

All participants provided informed, written consent to participate in the study, and the research protocol was approved by the regional scientific ethics committee (protocol number H-16047666).

### Blood samples

Peripheral blood mononuclear cells (PBMCs) were obtained from freshly collected venous blood using a density gradient centrifugation technique (Lymphoprep; Axis-Shield, Oslo, Norway). After isolation, PBMCs underwent two consecutive washes in cold phosphate-buffered saline (PBS) supplemented with 2-mM-ethylenediaminetetraacetic-acid (EDTA). Freshly isolated PBMCs were used for flow cytometry phenotyping, and excess PBMCs were cryopreserved in fetal bovine serum (FBS) with 10% dimethyl sulfoxide (DMSO) for subsequent *in vitro* analysis.

### Flowcytometry analysis of freshly isolated cells

A minimum of 350,000 freshly isolated PBMCs were incubated with an Fc receptor-blocking reagent (Miltenyi Biotec, Bergisch Gladbach, Germany) and the cells were stained with fluorochrome-conjugated antibodies specific for the surface molecules of interest. For B cells the following antibodies were used: CD19 (PerCP/Cy5.5; HIB19), CD27 (FITC; 323), CD38 (BV421, HIT2) and CD11c (PE/Cy7; Bu15), all from BioLegend (San Diego, CA, USA). For T cells, the following antibodies were used: CD3 (APC/Cy7; HIT3a), CD4 (PerCP/Cy5.5; RPA-T4), CD25 (PE; M-A251), CD127 (APC; A019D5), CXCR5 (AF488; J252D4), PD-1 (BV605; EH12.2H7), CXCR3 (PE/Cy7; G025H7) and CCR6 (BV421; G034E3) from BioLegend. Where applicable, corresponding isotype controls were used. Absolute counts of T and B cell subpopulations were measured using TruCount beads (BD Biosciences, Franklin Lakes, NJ, USA). Flow cytometry data acquisition was performed using a FACS Canto II flow cytometer (BD Biosciences), and data analysis was conducted using FlowJo software (Tree Star, Inc., Ashland, OR, USA).

### PBMC stimulation and intracellular staining

Cryopreserved PBMCs from 20 of the untreated patients with RRMS, 20 cladribine-treated patients at W52, 20 cladribine-treated patients at W96, and an additional 20 healthy controls were thawed and cultured for 48 hours in RPMI 1640 medium supplemented with penicillin and streptomycin (50 U/ml Gibco, Waltham, MA, USA) and 10% human AB serum (Invitrogen, Carlsbad, CA, USA). Cells were either left unstimulated or stimulated with CpG-ODN 2006 TLR9 agonist (InvivoGen, San Diego, CA, USA) at a concentration of 2.5 μg/ml. To assess B cell cytokine production, the CpG-stimulated and unstimulated PBMCs were subsequently incubated with phorbol-myristate-acetate (PMA; Sigma-Aldrich, St. Louis, MO, USA) at a concentration of 10 ng/ml, 0.5 μg/ml ionomycin (Sigma-Aldrich), and 5 μg/ml brefeldin A (Sigma-Aldrich), at 37°C and 5% CO_2_ for 4 hours. Following incubation, cells were surface stained with fluorochrome-conjugated anti-CD19 antibody (PE/Cy7; HIB19) and a live/dead stain. Subsequently, cells were fixed, permeabilized, and stained with anti-IL-10 (APC; JES3-19F1), anti-TGF-β (BV421; TW7-16B4) and anti-LTα (PE; 359-81-11) all from BioLegend. Flow cytometry data acquisition was performed using a FACS Canto II flow cytometer and analyzed using FlowJo software.

### FluoroSpot antigen reactivity assay

Low fluorescent PVDF FluoroSpot plates (Mabtech AB, Nacka Strand, Sweden) were activated with 35% ethanol for 1 minute and washed with sterile water. Plates were then coated with a dilution of 15 μg/ml IFN-γ, IL-13, IL-17 and IL-21 capture antibodies in sterile PBS supplied in Mabtech FluoroSpot kits (#3654-1-1) and incubated for 24 h at 4°C. After incubation, plates were washed in sterile PBS and blocked for 2 h at RT with RPMI-1640/5% human serum (Invitrogen). 250,000 PBMCs from each of 20 untreated patients with RRMS, 20 cladribine-treated patients with RRMS at W52, 20 cladribine-treated patients with RRMS at W96, and 20 healthy controls were added to each well in RPMI-1640/5% human serum with 30 µg/ml myelin basic protein (MBP; HyTest, Turku, Finland), 10 µg/ml myelin oligodendrocyte glycoprotein (MOG; AnaSpec, Fremont, CA, USA), 0.3 µg/ml RAS guanyl releasing protein 2 (RASGRP2; OriGene Technologies Inc., Rockville, MD, USA), 10 µg/ml alpha B crystallin (CRYAB; Abcam, Cambridge, United Kingdom), 5x10^6^ cells/ml heat-killed Candida albicans (HKCA; InvivoGen) or medium alone. A costimulatory monoclonal anti-CD28 antibody (Mabtech) was added to the wells in a concentration of 0.1 µg/ml and plates were incubated for 48 h at 37°C, 5% CO_2_. Subsequently, cells were removed, and plates were washed with PBS and biotinylated-detection antibody in PBS/0.1% bovine serum albumin (BSA) was added for 2 h, at RT. Plates were washed and incubated for 1 h at RT with fluorophore-conjugated antibodies in PBS/0.1% BSA according to manufacturer (Mabtech AB). Subsequently plates were incubated with fluorescence enhancer for 15 minutes and afterwards emptied and left to dry protected from daylight for a minimum of 45 minutes. Analysis of spots was performed using the Mabtech IRIS reader system equipped with Apex™ software (Mabtech AB).

The included untreated patients with RRMS and HC were selected to match the group of cladribine treated patients with RRMS. There were no statistically significant differences in age or sex between any of the included groups.

### Statistics

Statistical analyses were performed using GraphPad Prism 7 software (GraphPad software Inc, La Jolla, CA, USA). Mann Whitney U test was applied for comparison of B and T cell subpopulations, between untreated patients with RRMS and patients treated with cladribine for 52 weeks. Friedman paired test was applied for comparison of B and T cell subpopulations between W52, W60, W72 and W96 of cladribine treatment. Kruskal-Wallis test was applied for comparison of B cell cytokine production and CNS antigen reactivity between untreated patients with RRMS and W52 and W96 of cladribine treatment where a Mann Whitney U test was applied for comparison between healthy controls and untreated patients with RRMS. A p value <0.005 was considered statistically significant and <0.05 as suggestive of significance (37).

### STROBE guidelines

For this manuscript the STROBE reporting guidelines for observational studies was used.

### Data availability

Data are available in anonymized form and can be shared by request form any qualified investigator. Sharing requires approval of a data transfer agreement by the Danish Data Protection Agency.

## Results

### Patient characteristics

We included 30 untreated patients with RRMS, and 20 patients treated with oral cladribine tablets (**Table 1**). The two groups showed no significant differences in age, sex, or EDSS scores. However, patients in the cladribine group had a longer disease duration (p<0.0001). Among the untreated patients, all had experienced either relapse or magnetic resonance imaging (MRI) activity in the previous year. In the cladribine-treated group, seven had neither relapse nor MRI activity in the year prior to initiating cladribine therapy. These patients had switched from another treatment due to side effects or the development of anti-JC virus antibodies. In the cladribine group, three patients had not received any previous treatment, while 12 had received oral therapies (teriflunomide, dimethyl fumarate, or fingolimod), and five had received monoclonal antibody treatment (natalizumab or rituximab). Before initiating cladribine treatment, all previously treated patients had normal lymphocyte counts, except for one patient previously treated with dimethyl fumarate who had grade 1 lymphopenia.

**Table 1:** Patient characteristics.

	Untreated RRMS (n = 30)	CLA (n = 20)
**Biological sex, male/female**	8 (27%), 22 (73%)	8 (40%), 12 (60%)
**Age, years**	35 [21-62]	41 [27-59]
**Disease duration, years**	1 [0-25]	9 [1-31]
**EDSS Score**	1.75 [0-4]	2.5 [0-6]
**Relapse previous year**	27/30 (90%)	12/24 (50%)
**MRI activity without** **relapse previous year**	3/3 (100%)	5/12 (42%)
**Previous therapy**	None (n=30, 100%)	None (n=3, 15%) Teriflunomide (n=3, 13%) Dimethyl fumarate (n=5, 21%) Fingolimod (n=4, 20%) Natalizumab (n=4, 20%) Rituximab (n=1, 4%)

RRMS, relapsing-remitting multiple sclerosis; CLA, cladribine treated patients with RRMS; EDSS, expanded disability status scale; MRI, magnetic resonance imaging; n, number of individuals. Data are given as numbers and percentages or median and range.

### Cladribine-treatment induces long-term memory B cell suppression

**Figure 1:**
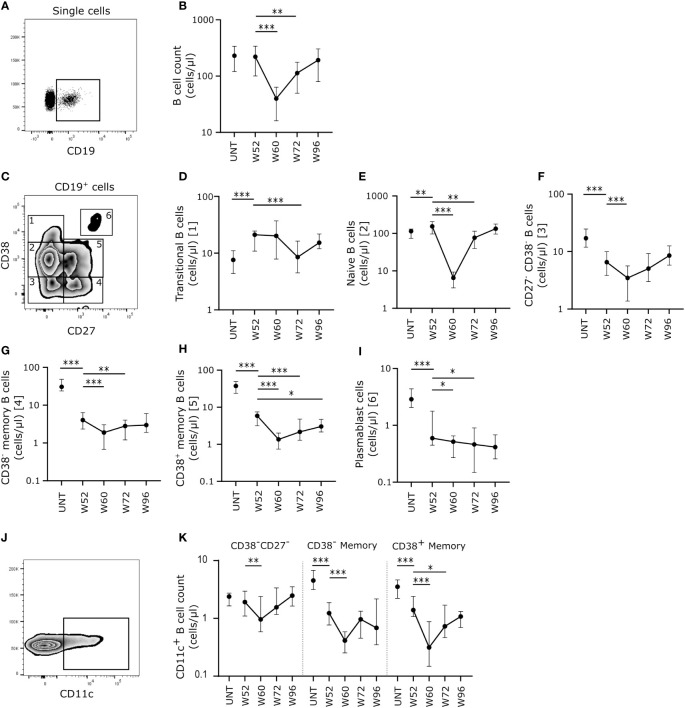
Cladribine-treatment induce long-term memory B cell suppression. **(A)** Gating strategy of CD19^+^ B cells. Gating strategy includes a CD19^+^ gate in a dot plot of single cell lymphocytes. **(B)** Scatterplots showing absolute counts (cells/μl blood) of total CD19^+^ B cells. **(C)** Gating strategy of CD19^+^ B cell substes. Gating strategy includes a CD27/CD38 zebra plot for gating of the six different B cell subtypes in blood; 1: CD27^-^CD38^++^ transitional B cells, 2: CD27^-^CD38^+^ naïve B cells, 3: CD27^-^CD38^-^ B cells, 4: CD27^+^CD38^-^ memory B cells, 5: CD27^+^CD38^+^ memory B cells and 6: CD27^++^CD38^++^ plasmablasts. **(D–I)** Scatterplots showing absolute counts (cells/μl blood) transitional B cells **(D)**, naïve B cells **(E)**, CD27^-^CD38^-^ B cells **(F)**, CD27^+^CD38^-^ memory B cells **(G)**, CD27^+^CD38^+^ memory B cells **(H)** and plasmablasts **(I)** from untreated patients with RRMS (UNT; n=30) and patients treated with cladribine for 52 (W52), 60 (W60), 72 (W72) and 96 (W96) weeks, n=20. **(J)** A flow cytometry zebra-plot example of CD11c gating on memory B cells. **(K)** A scatterplot showing absolute counts (cells/μl blood) of CD11c^+^ CD27^-^CD38^-^ B cells, CD27^+^CD38^-^ memory B cells and CD27^+^CD38^+^ memory B cells from untreated patients with RRMS (UNT; n=30) and patients treated with cladribine for 52 (W52), 60 (W60), 72 (W72) and 96 (W96) weeks, n=20. The median value is shown for all groups analyzed. ***p < 0.0001; **p < 0.005; *p < 0.05.

We found no difference in absolute count of CD19^+^ B cells between 30 untreated patients with RRMS and 20 patients treated with cladribine at W52 (**Figures 1A, B**). However, at W60, i.e., eight weeks after the initiation of the second treatment cycle we found a significantly lower number of CD19^+^ B cells, that was partly maintained to W72, but at W96 CD19^+^ B cell levels were reconstituted to levels comparable to W52 (**Figure 1B**). When analyzing the specific B cell subsets according to their expression of CD27 and CD38 (**Figure 1C**) the number of transitional (**Figure 1D**) and naïve (**Figure 1E**) B cells were higher in patients treated with cladribine at W52 compared to untreated, and although there was a significant decrease in numbers of transitional B cells at W72 and naïve B cells at W60 and W72, W96 numbers were comparable to W52 numbers.

Conversely, looking at the memory B cell compartment we found a significantly lower number of CD27^-^CD38^-^ (**Figure 1F**), CD38^-^ (**Figure 1G**) and CD38^+^ memory (**Figure 1H**) B cells and plasmablasts (**Figure 1I**) at W52 of cladribine treatment compared to untreated patients. We also found that the number of all memory B cell subsets were significantly lower at W60 and W72 compared to W52, except for CD27^-^CD38^-^ memory B cells that only showed a decrease in numbers at W60 and the CD38^+^ memory B cells which remained significantly lower at W96 than at W52.

Investigating atypical, CD11c^+^ memory B cells (**Figure 1J**) showed that at W52 of cladribine treatment there were significantly lower numbers of CD11c^+^CD38^-^ and CD38^+^ memory B cells whereas no difference was observed for CD38^-^CD27^-^ B cells compared to untreated patients with RRMS (**Figure 1K**). In all three CD11c^+^ B cell memory subpopulations there was a significant decrease in numbers at W60 and for the CD38^+^ memory B cells the decrease persisted at W70, but at W96 of cladribine treatment CD11c^+^ memory B cells were reconstituted to levels comparable to W52 (**Figure 1K**).

### Long-term cladribine treatment induces a shift in Tfh: Tfr cell ratio

**Figure 2:**
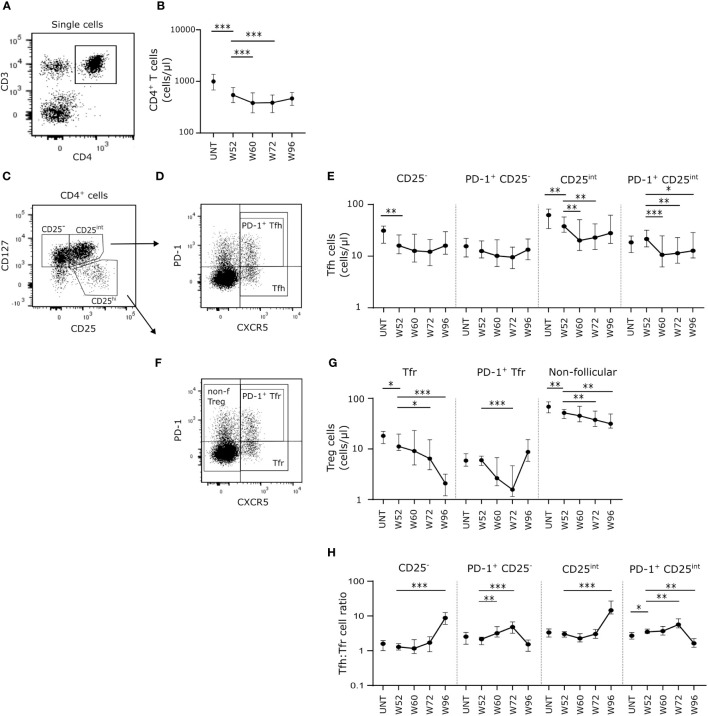
Long-term cladribine treatment induces a shift in Tfh: Tfr cell ratio. **(A)** Gating strategy of CD4^+^ T cells. Gating strategy includes a CD3/CD4 gate in a dot plot of single cell lymphocytes. **(B)** Scatterplots showing absolute counts (cells/μl blood) of total CD4^+^ T cells. **(C, D, F)** Gating strategy of Tfh, Tfr and non-follicular Treg cells. Gating strategy includes a CD127/CD25 dot plot for gating of CD127^+^CD25^-^, CD127^+^CD25^int^ and CD127^-^CD25^hi^ **(C)**, and a PD-1/CXCR5 dot plot for PD-1^+/-^Tfh **(D)**, PD-1^+/-^ Tfr and non-follicular Treg cells **(F)**. **(E–G)** Scatterplots showing absolute counts (cells/μl blood) of CD25^-^ Tfh cells, PD-1^+^CD25^-^ Tfh cells, CD25^int^ Tfh cells and PD-1^+^CD25^int^ Tfh cells **(E)** and Tfr cells, PD-1^+^ Tfr cells and non-follicular Treg cells **(G)** from untreated patients with RRMS (UNT; n=30) and patients treated with cladribine for 52 (W52), 60 (W60), 72 (W72) and 96 (W96) weeks, n=20. **(H)** Scatterplots showing Tfh: Tfr cell ratios of CD25^-^, PD-1^+^CD25^-^, CD25^int^ and PD-1^+^CD25^int^ Tfh: Tfr from untreated patients with RRMS (UNT; n=30) and patients treated with cladribine for 52 (W52), 60 (W60), 72 (W72) and 96 (W96) weeks, n=20. The median value is shown for all groups analyzed. ***p < 0.0001; **p < 0.005; *p < 0.05.

Cladribine treatment induced long-term depletion of CD4^+^ T cells persisting from W52 to W96 with even lower numbers of circulating cells at W60 and W72 than W52 (**Figures 2A, B**). We measured the absolute count of Tfh and Tfr cells and non-follicular regulatory T cells (Treg) in order to investigate the B cell activation potential. We defined Tfh cells as CD4^+^CD127^+^CXCR5^+^, Tfr cells as CD4^+^CD127^-^CD25^hi^CXCR5^+^, activated Tfh and Tfr cells as PD-1^+^ and non-follicular Tregs as CD4^+^CD127^-^CD25^hi^CXCR5^-^ T cells (**Figures 2C, D, F**). The number of circulating CD25^-^ and CD25^int^ Tfh cells was significantly lower at W52 compared to untreated patients with RRMS (**Figure 2E**). While the number of CD25^-^ Tfh cells was unchanged from W52 to W96, the number of CD25^int^ Tfh cells decreased significantly at W60 and W72 (Figure E). There were no changes in numbers of PD-1^+^ Tfh cells between untreated patients with RRMS and W52 of cladribine treatment, but after the second treatment cycle PD-1^+^ CD25^int^ Tfh cells were significantly reduced at W60, W72 and W96 compared to W52 (**Figure 2E**).

Looking at the regulatory potential we found a suggestively significant lower number of Tfr cells at W52 in cladribine-treated patients compared to untreated, and furthermore we found a suggestively significant decrease in the number of Tfr cells at W72 and a significantly lower number after W96 compared to W52 in the cladribine-treated patients (**Figure 2G**). There were no changes in numbers of PD-1^+^ Tfr cells between untreated and W52 of cladribine-treated patients with RRMS. There was a significantly lower number of PD-1^+^ Tfr cells at W72 compared to W52 of cladribine treatment, but at W96 the number of PD-1^+^ Tfr cells were reconstituted to levels comparable to W52 (**Figure 2G**). Total numbers of non-follicular Tregs were lower at W52 in cladribine-treated patients compared to untreated and significantly lower at W72 and W96 of cladribine treatment than at W52 (**Figure 2G**).

Analyzing the Tfh: Tfr ratio between groups showed increased Tfh: Tfr cell ratios of both CD25^-^ and CD25^int^ cells at W96 compared to W52 in cladribine-treated patients. Likewise, both PD-1^+^CD25^-^ and PD-1^+^CD25^int^ Tfh: Tfr cell ratios were higher at W72 compared to W52. However, at W96 of cladribine therapy we found a shift in PD-1^+^CD25^int^ Tfh: Tfr cell ratios with a significantly lower ratio compared to W52 (**Figure 2H**).

### Long-term cladribine treatment induces lower numbers of proinflammatory effector T cells

**Figure 3:**
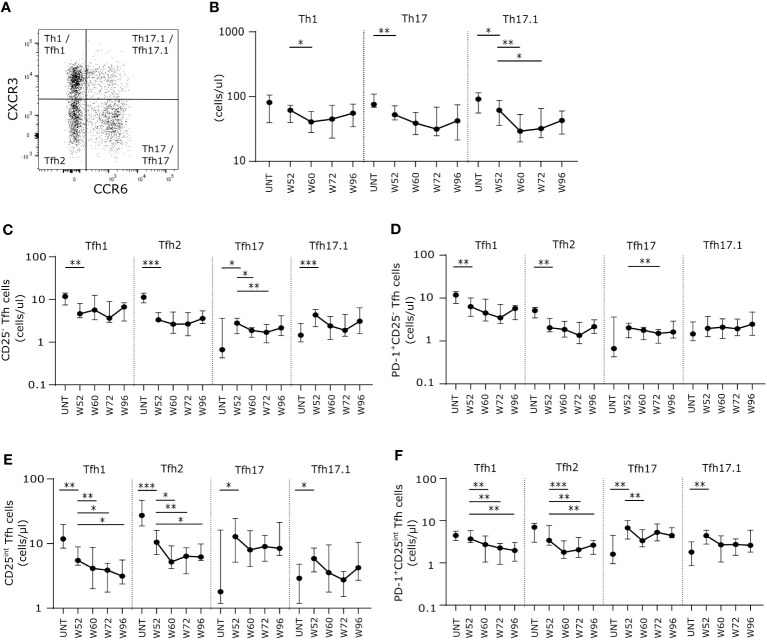
Long-term cladribine treatment induce lower numbers of proinflammatory effector T cells and changes in Tfh effector subsets. **(A)** Gating strategy of Th1, Th17, Th17.1, Tfh1, Tfh2, Tfh17 and Tfh17.1. Gating strategy includes a CXCR3/CCR6 gate in a dot plot of CXCR5^-^ or CXCR5^+^ CD4^+^CD127^+^ T cells. **(B)** Scatterplots showing absolute counts (cells/μl blood) of non-follicular CXCR5^-^ Th1, Th17 and Th17.1. **(C–F)** Scatterplots showing absolute counts (cells/μl blood) of Tfh1, Tfh2, Tfh17 and Tfh17.1 cells within CD25^-^ Tfh cells **(C)**, PD-1^+^CD25^-^ Tfh cells **(D)**, CD25^int^ Tfh cells **(E)** and PD-1^+^CD25^int^ Tfh cells **(F)** from untreated patients with RRMS (UNT; n=30) and patients treated with cladribine for 52 (W52), 60 (W60), 72 (W72) and 96 (W96) weeks, n=20. The median value is shown for all groups analyzed. ***p < 0.0001; **p < 0.005; *p < 0.05.

Analyzing the long-term cladribine-induced effects on non-follicular effector T cells (CD4^+^CD127^+^CXCR5^-^), we measured absolute counts of the functionally distinct effector T cells subsets Th1, Th17 and Th17.1 characterized according to their expression of CXCR3 and CCR6 (**Figure 3A**). After W52 of cladribine treatment we found a significant and suggestively significant lower number of Th17 and Th17.1 cells, respectively, compared to untreated patients with RRMS. (**Figure 3B**). After the second cladribine treatment cycle we found a suggestively significant decrease in Th1 cells at W60 compared to W52, however, at W72 and W96 Th1 cell numbers were comparable to W52. (**Figure 3B**). Likewise, numbers of Th17.1 were significantly lower at W60 and W72 but reconstituted at W96 to levels comparable to W52 (**Figure 3B**).

### Long-term cladribine treatment induces changes in Tfh subsets

We further subdivided the Tfh cell subsets into the effector subpopulations Tfh1 (CXCR3^+^CCR6^-^), Tfh2 (CXCR3-CCR6-), Tfh17 (CXCR3^-^CCR6^+^) and Tfh17.1 (CXCR3^+^CCR6^+^) (**Figure 3A**). Here we found that for both CD25^-^, PD-1^+^CD25^-^ and CD25^int^ Tfh cell populations the number of Tfh1 and Tfh2 cells was significantly lower at W52 in cladribine-treated patients compared to untreated patients (**Figures 3C–F**). Conversely, numbers of Tfh17 cells were higher in both the CD25^int^ and the PD-1^+^CD25^int^ Tfh cell populations, and Tfh17.1 cells were significantly higher in CD25^-^ and PD-1^+^CD25^int^ Tfh cells at W52 in cladribine-treated patients compared to untreated (**Figures 3C–F**). After the second treatment cycle numbers of CD25^-^, PD-1^+^CD25^-^ and PD-1^+^CD25^int^ Tfh17 cells were lower at W60 or W72 compared to W52 but were reconstituted to levels comparable to W52 at W96 (**Figures 3C–F**). Conversely, numbers of CD25^int^ and PD-1^+^CD25^int^ Tfh1 and Tfh2 cells were significantly lower at both W60, W72 and W96 compared to W52 of cladribine treatment (**Figures 3C–F**).

### Long-term cladribine treatment induces lower frequencies of LTα producing B cells

**Figure 4:**
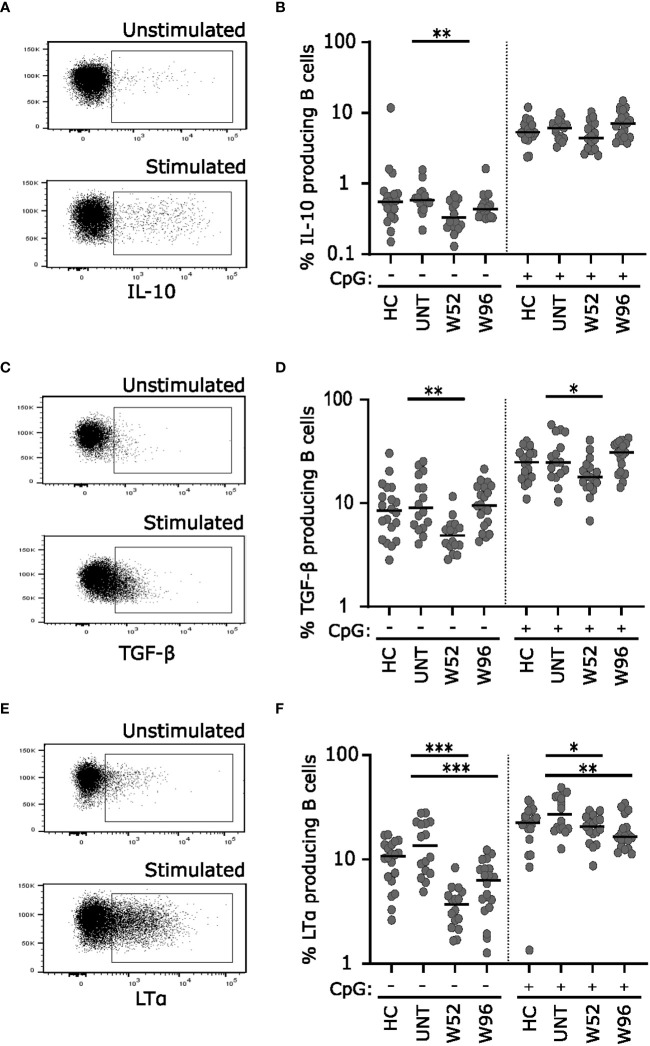
Long-term cladribine treatment induces lower frequencies of LTα producing B cells. Dot plot gating examples of intracellular IL-10 **(A)**, TGF-β **(C)** and LTα **(E)** staining of B cells. The *upper* panel shows unstimulated cells and the *lower* panel show cells stimulated for 48 h with CpG followed by a short re-stimulation. Scatterplots showing frequencies of IL-10 **(B)** TGF-β **(D)** and LTα **(F)** producing B cells from healthy controls (HC; n=20), untreated patients with RRMS (UNT; n=20) and patients treated with cladribine for 52 (W52) and 96 (W96) weeks, n=20. The median value is shown for all groups analyzed. ***p < 0.0001; **p < 0.005; *p < 0.05.

To analyze the long-term effects of cladribine treatment on B cell cytokine responses, we stimulated cryopreserved PBMCs from 20 healthy controls (median age 42 years; range 24-69 years), 20 untreated patients with RRMS (median age 37 years; range 26-62 years) and 20 patients treated with cladribine at W52 and W96 with or without CpG for 2 days and evaluated the IL-10, TGF-β, and LTα profile (**Figures 4A, C, E**) of B cells after a short re-stimulation. (**Figures 4B, D, F**) Without CpG-stimulation we found a significantly lower frequency of IL-10, TGF-β, and LTα producing B cells at W52 compared to untreated patients, however, frequencies of both IL-10 and TGF-β producing B cells were reconstituted to levels comparable to untreated patients at W96. Following CpG stimulation there were no differences between frequencies of IL-10 producing B cells in any of the groups, but frequencies of TGF-β were suggestively significantly decreased compared to untreated at W52 but not at W96 (**Figures 4B, D**). Conversely, the frequency of LTα producing B cells remained lower in cladribine-treated than in untreated patients at W52 and W96 with and without CpG stimulation (**Figure 4F**).

### Long-term cladribine treatment induces lower reactivity towards MOG, MBP, CRYAB and RASGRP2

**Figure 5:**
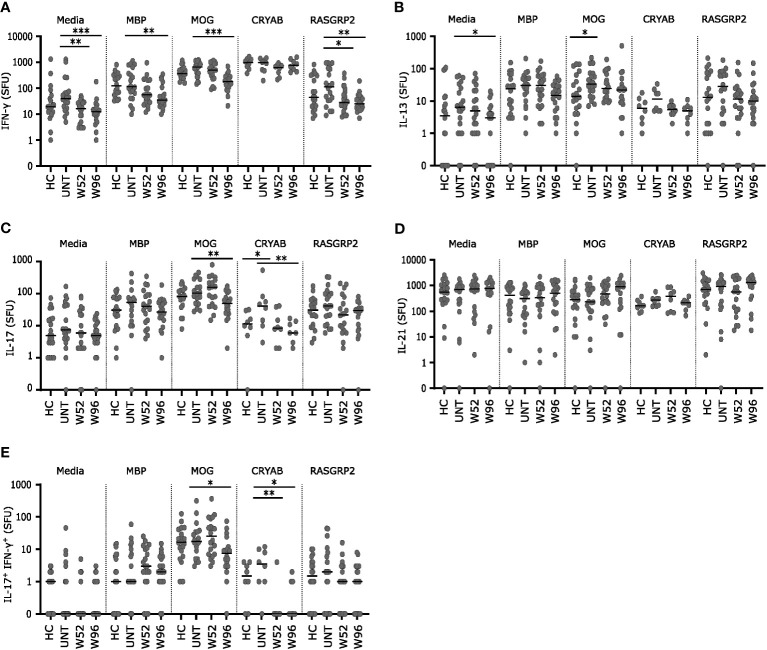
Long-term cladribine treatment induce lower reactivity towards MOG, MBP, CRYAB and RASGRP2. **(A–E)** Scatterplots showing spot forming units (SFU) of IFN-γ **(A)**, IL-13 **(B)**, IL-17 **(C)**, IL-21 **(D)** and IL-17^+^IFN-γ^+^ **(E)** reactivity against media, myelin basis protein (MBP), myelin oligodendrocyte glycoprotein (MOG), alpha B crystallin (CRYAB; n=8 from each group) and RAS guanyl releasing protein 2 (RASGRP2) from PBMCs from healthy controls (HC; n=20), untreated patients with RRMS (UNT; n=20) and patients treated with cladribine for 52 (W52) and 96 (W96) weeks, n=20. The median value is shown for all groups analyzed. ***p < 0.0001; **p < 0.005; *p < 0.05.

Cryopreserved PBMCs from 20 healthy controls, 20 untreated patients with RRMS and 20 patients treated with cladribine at W52 and W96 were stimulated for 48 h with the autoantigens MOG, MBP, CRYAB (for 8 patients from each group), or RASGRP2 together with co-stimulatory anti-CD28 or medium with anti-CD28 alone as control. Following stimulation, IFN-γ, IL-17, IL-13 and IL-21 spot forming units (SFU) at the single cell level were measured. Untreated patients with MS had a suggestively higher number of IL-13 and IL-17 SFUs than healthy controls after stimulation with MOG and CRYAB respectively (**Figures 5B, C**).

IFN-γ and IL-13 SFUs were lower after stimulation in medium with anti-CD28 at W96 and for IFN-γ SFUs also at W52 compared to untreated patients (**Figures 5A, B**). IFN-γ SFUs were significantly lower at W96 in response to MBP, MOG and RASGRP2 and the IFN-γ response to RASGRP2 was also suggestively significantly lower at W52 compared to untreated (**Figure 5A**). IL-17 SFUs were significantly lover at W96 in response to MOG and CRYAB compared to untreated patients (**Figure 5C**) IFN-γ^+^IL-17^+^ SFUs were significantly lower in response to CRYAB at W52 and furthermore suggestively significant lower at W96 in response to both CRYAB and MOG compared to untreated patients (**Figure 5E**). There were no difference in IL-21 response between groups to any of the autoantigens (**Figure 5D**).

## Discussion

Several studies, including a recent study from our group, have investigated the effects of cladribine on circulating immune cells during the first year of treatment (27–34). In the present study we follow up on 20 of the 24 patients included in our previous study with a detailed analysis of changes in circulating immune cell phenotypes, B cell cytokine production profiles, and autoantigen reactivity during the second year of treatment (35), i.e., after the administration of a full treatment course of 3.5 mg cladribine/kg body weight (23).

In the immune cell phenotype studies, we focused on B cell subsets and function, and confirmed that cladribine treatment has long-term effects on memory B cells. Compared to W52, total B cell counts decreased at W60 and W72 but had returned to normal levels at W96, consistent with the results of other studies investigating cladribine effects over 2 years (28, 30, 32, 33). Numbers of transitional and naïve B cells, memory B cells and plasmablasts decreased at W60 and/or W72 compared with W52. However, whereas numbers of circulating plasmablasts, CD27^-^CD38^-^ B cells and CD38^-^ and CD38^+^ memory B cells were lower than in untreated patients, numbers of transitional and naïve B cells were higher at W52 and W96. There were no significant differences between B cell reconstitution at W52 and W96, consistent with the results of a previous study (33).

We also found that the numbers of CD38^-^ and CD38^+^ memory B cells expressing CD11c were lower at W52 and W96 than in untreated patients and decreased further from W52 to W60 and/or W72. CD11c^+^ B cell were previously shown to produce proinflammatory cytokines and develop into autoantibody-producing cells (38). We studied induced B cell cytokine production with or without priming with CpG, which stimulates Toll-like receptor 9, before a brief stimulation with PMA and ionomycin (18). At W52 we observed a lower percentage of B cells expressing IL-10 without priming with CpG whereas there was no difference at W96, and there was no difference in the percentage of B cells expressing IL-10 when the cells had been primed with CpG. For B cells expressing TGF-β we found a lower percentage at W52 both with and without priming with CpG. However, at W96 there was no difference in TGF-β expression between treated and untreated patients. We also found that at W52 and W96 the percentage of B cells producing LT-α was lower both with and without priming with CpG in cladribine-treated patients than in untreated patients. Taken together, these data support the notion that cladribine-treated patients are characterized by long-term depletion of proinflammatory memory B cells and that although a decrease in the production of the immunoregulatory cytokines IL-10 and TGF-β was observed under some culture conditions at W52, this was not observed at W96, whereas a consistent decrease in the production of the proinflammatory cytokine LT-α was observed both at W52 and W96. A dysregulation of IL-10 and LTα has been shown in patients with MS with a higher percentage of B cells producing LTα (18, 19) and a lower percentage of B cells producing IL-10 (17, 19) and these imbalances have been suggested to contribute to exaggerated Th1 and Th17 responses in MS (19).

Consistent with previous studies we find that the depletion of circulating CD4^+^ T cells is maintained one year after the second treatment cycle (28, 29). We investigated effector T cell subsets involved in the activation of B cells, i.e., follicular helper T (Tfh) cells and follicular regulatory T (Tfr) cells. One year after the first cladribine treatment cycle the total number of Tfh cells was significantly lower than in untreated patients, and this decrease was maintained or even lower in the second year of treatment. We subdivided the Tfh cells into a CD25^-^ (Th1-like) and a CD25^int^ (Th17-like) subset and used expression of PD-1 as a marker of Tfh activation (15). Numbers of PD-1^-^CD25^-^ Tfh and PD-1^+^ and PD-1^-^CD25^int^ Tfh cells were lower in patients treated with cladribine, and the number of PD-1^+^CD25^int^ Tfh cells decreased even further from W52 to W96. We did, however, also find that the total number of Tfr cells was lower at W52 and although the number of PD-1^+^ Tfr cells was higher at W96, the total number of Tfr cells was even lower at W96 than at W52.

A skewing in the Tfh: Tfr cell ratio in favor of Tfh cells has previously been observed in patients with RRMS (11, 39). One year after the first cladribine treatment cycle we found a suggestively significant higher PD-1^+^CD25^int^ Tfh: Tfr cell ratio compared to untreated patients, suggesting that the functional activity of Tfh cells is maintained or even increased in cladribine-treated patients. After the second treatment cycle the PD-1^+^ Tfh: Tfr cell ratio was initially increased at W72 but at W96 we found a shift in the PD-1^+^ CD25^int^ Tfh: Tfr cell ratio with significantly lower ratio compared to W52, suggesting a return of the regulatory function after the second cladribine treatment cycle.

In a separate analysis we used expression of the chemokine receptors CCR6 and CXCR3 to identify Tfh1 (CXCR3^+^CCR6^-^), Tfh2 (CXCR3^-^CCR6^-^), Tfh17 (CXCR3^-^CCR6^+^) and Tfh17.1 (CXCR3^+^CCR6^+^) cells. Tfh17 cells induce naïve human B cells to secrete IgA, IgG and IgM, and Tfh2 cells induce the secretion of IgG, IgA and IgM as well as IgE (15). Both in the CD25^-^ and the CD25^int^ Tfh subset and in PD-1 positive and negative Tfh cells there was an increase in the absolute number of Tfh17 cells in blood in patients treated with cladribine. Although transient decreases in numbers were observed at W60 or W72 for some subsets, overall Tfh17 cells remained at higher levels than in untreated patients throughout year 2 of cladribine treatment. In contrast, the numbers of Tfh2 cells were lower in all Tfh subsets, and even decreased further from W52 to W96 for some subsets. We hypothesize that the increased number of circulating Tfh17 cells may compensate for the changes in other Tfh subsets and Tfh: Tfr ratios and help explain why patients treated with cladribine maintain normal immunoglobulin levels and preserved humoral responses to vaccination in spite of lower numbers of circulating memory B cells and plasmablasts (27, 31, 34, 40).

Overall, we found lower counts of circulating Tfh1 cells in patients treated with cladribine than in untreated patients and for some subsets the counts were even lower at W96 than at W52. In contrast to Tfh2 and Tfh17 Tfh cells, Tfh1 Tfh cells do not support the differentiation of naïve B cells to immunoglobulin-secreting cells. This may reflect that CXCR3 expression counteracts the retention of Tfh cells in germinal centers (41). We are not aware of studies addressing the Tfh activity of Tfh17.1 cells but since these cells also express CXCR3 they may also have poor Tfh activity, and we found only minor effects on Th17.1 Tfh cells in patients treated with cladribine. The role of changes in Tfh1 and Tfh17.1 cells in patients treated with cladribine therefore remains to be established.

In addition to lower numbers of total CD4^+^ T cells, there were lower numbers of Th1, Th17 and Th17.1 cells at W52 and W96, and there was a further decrease in Th17.1 cells at W60 and W72 compared with untreated patients. Th1, Th17 and Th17.1 cells have all been implicated in the pathogenesis of MS (2, 42). We did, however, also find that the number of circulating non-follicular Treg cells was lower at W52 and decreased further during the second year of treatment with cladribine. This reduction in Treg cells may help preserve protective T cell responses in patients treated with cladribine.

We also investigated the cytokine responses of circulating immune cells in patients treated with cladribine. When cells were cultured in control medium with anti-CD28 alone there were fewer cells secreting IFN-γ at W52 and 96 and fewer cells secreting IL-13 at W96 compared to untreated patients with RRMS. Using MOG as antigen there were more cells secreting IL-13 in untreated patients with MS than in healthy controls. There was no significant difference in the number of MOG-reactive cells secreting IFN-γ, IL-21 or cells secreting both IFN-γ and IL-17A (Th17.1 cells) between untreated patients and healthy controls, and there were no differences between untreated patients and healthy controls in autoreactive cells after stimulation with MBP, CRYAB or RASGRP2. Increased T cell responses to MBP and RASGRP2 were reported in MS in previous studies (43–46). The use of anti-CD28 for co-stimulation in the present study may at least partly explain this difference, and this may also explain why there was substantial reactivity to all autoantigens even in the control subjects. Furthermore, the results on CRYAB reactivity should be interpreted with caution since insufficient supplies resulted in the antigen only being studied in some of the subjects.

At W52 the IFN-γ response to RASGRP2 was lower than in untreated patients, as also observed in our previous study (35). At W96 the IFN-γ response to RASGRP2 was still reduced and there were also lower responses to MBP and MOG. We also found that the Th17 and Th17.1 (IL-17A^+^ and IFN-γ^+^IL-17A^+^) response to CRYAB was lower in patients treated with cladribine both at W52 and W96 than in untreated patients. It has been suggested that memory B cells expressing RASGRP2 can initiate and propagate an autoreactive T cell response, leading to CNS migration and causing chronic brain inflammation (43, 45). The autoantigen CRYAB was originally identified as a small heath shock protein present in the cytosol of oligodendrocytes and astrocytes in MS lesions (47). Later it was shown that Epstein Barr virus infection induced CRYAB expression in B cells, and T cells reactive to CRYAB were identified both in healthy controls and patients with MS (47, 48). Our observation of lower Th1 responses to RASGRP2 and lower Th17.1 responses to CRYAB at W52 and W96 is consistent with our hypothesis that lower numbers of circulating memory B cells may lower T cell reactivity to autoantigens expressed in B cells. The lower reactivity to MBP and MOG after 96 weeks does, however, suggest that other mechanisms, including a general decrease in IFN-γ reactivity may contribute. We did, indeed, observe lower numbers of unstimulated cells secreting IFN-γ at W52 and W96, but we only observed lower reactivity to MOG and MBP at W96.This may also, at least partly, explain why patients treated with cladribine are at increased risk of certain viral infections which are generally controlled by IFN-γ-dependent type 1 immune responses (49).

We conclude that the effects of cladribine observed after year one are maintained and, for some effects, even increased two years after the initiation of a full course of treatment with cladribine tablets. The most pronounced effect was a substantial reduction in circulating memory B cells and proinflammatory B cell responses. This is consistent with a reduction of memory B cells being a general feature of disease-modifying MS therapies (50). Furthermore, we observed reduced T cell responses to autoantigens presented by B cells at W52 and a further reduction in responses to the myelin antigens MBP and MOG after 96 weeks. We cannot exclude that the effects of previous treatment could contribute as only few patients in our study were previously untreated but our results compare well with the results of the MAGNIFY-MS study where half of the patients were previously untreated. However, neither the MAGNIFY-MS study nor any other previous studies of which we are aware compared the immunological effects of cladribine in previously treated and untreated patients. Future studies are needed to confirm these observations in independent patient cohorts and address the possible impact of previous treatments on the response to cladribine. Furthermore, it should be addressed whether differences between patients in the magnitude of these effects are associated with the effectiveness of treatment with cladribine tablets.

## Data availability statement

The raw data supporting the conclusions of this article will be made available by the authors, without undue reservation.

## Ethics statement

The studies involving humans were approved by The regional scientific ethics committee (protocol number H-16047666). The studies were conducted in accordance with the local legislation and institutional requirements. The participants provided their written informed consent to participate in this study.

## Author contributions

RH: Conceptualization, Data curation, Formal analysis, Funding acquisition, Investigation, Methodology, Project administration, Resources, Software, Validation, Visualization, Writing – original draft, Writing – review & editing. MV: Conceptualization, Funding acquisition, Supervision, Writing – review & editing. MR: Data curation, Investigation, Writing – review & editing. SC: Data curation, Investigation, Writing – review & editing. FS: Conceptualization, Funding acquisition, Methodology, Project administration, Resources, Supervision, Writing – review & editing.
